# Measuring Agarwood Formation Ratio Quantitatively by Fluorescence Spectral Imaging Technique

**DOI:** 10.1155/2015/205089

**Published:** 2015-05-18

**Authors:** Botao Huang, Duykien Nguyen, Tianyi Liu, Kaibin Jiang, Jinfen Tan, Chunxin Liu, Jing Zhao, Shaowei Huang

**Affiliations:** ^1^College of Science, South China Agricultural University, Guangzhou, Guangdong 510640, China; ^2^Guangdong Key Laboratory for Innovative Development and Utilization of Forest Plant Germplasm, South China Agricultural University, Guangzhou, Guangdong 510640, China; ^3^College of Forestry, South China Agricultural University, Guangzhou, Guangdong 510640, China

## Abstract

Agarwood is a kind of important and precious traditional Chinese medicine. With the decreasing of natural agarwood, artificial cultivation has become more and more important in recent years. Quantifying the formation of agarwood is an essential work which could provide information for guiding cultivation and controlling quality. But people only can judge the amount of agarwood qualitatively by experience before. Fluorescence multispectral imaging method is presented to measure the agarwood quantitatively in this paper. A spectral cube from 450 nm to 800 nm was captured under the 365 nm excitation sources. The nonagarwood, agarwood, and rotten wood in the same sample were distinguished based on analyzing the spectral cube. Then the area ratio of agarwood to the whole sample was worked out, which is the quantitative information of agarwood area percentage. To our knowledge, this is the first time that the formation of agarwood was quantified accurately and nondestructively.

## 1. Introduction

Agarwood (*Aquilaria sinensis* Lour. Gilg) is a dark resinous heartwood that forms in* Aquilaria* trees, which was distributed in South China such as Hainan province, Guangxi province, and Guangdong province [1–3]. It forms when* Aquilaria* trees are infected with a type of mould. Prior to infection, the heartwood is relatively light and pale coloured; however, as the infection progresses, the tree produces a dark aromatic resin in response to the attack, which results in a very dense, dark, resin embedded heartwood [4].

Agarwood is valued in many cultures for its distinctive fragrance and thus is used for incense and perfumes. It is also known as a kind of famous traditional Chinese medicine. It can be used as sedative, analgesic, and digestive medicine in the orient [5–7]. Moreover, it had been reported that agarwood can help healing rheumatism, cardiovascular and cerebrovascular diseases, and many other diseases related to the artery and the heart [8, 9].

One of the main reasons for the relative rarity and high cost of agarwood is the depletion of the wild resource. Since 1995,* Aquilaria malaccensis*, the primary source, has been listed in potentially threatened species by the Convention on International Trade in Endangered Species of Wild Fauna and Flora. In 2004 all* Aquilaria* species were listed in potentially threatened species. For China, it had been claimed in China Plant Red Data Book in 1992 that, because of the deforestation of the nonagarwood which can generate the agarwood and lead to the rare situation of agarwood now, the way of cultivating should be the main method to get agarwood [10]. Nowadays the most common way of cultivating the agarwood is the Hong inoculated knot [2, 11]. For better quality control of agarwood cultivation, it is essential to choose the suitable* Aquilaria *trees. There are several factors to show which trees are suitable, of which agarwood formation ratio is one of the most significant factors. Agarwood formation is the ratio of agarwood cross-sectional area to tree cross-sectional area. The larger the area of the agarwood is, the better the tree is for formation agarwood. So it is necessary to test the area of the agarwood in different trees and then use the statistical data to determine which is the more suitable tree. It is essential and important for selective breeding and evaluating quality of agarwood. However, there is no effective method to measure the area except rough estimation by human eyes. In this paper, the technology of fluorescence spectral imaging was presented to measure the area of the agarwood. As far as we know, this is the first time that the agarwood formation ratio was measured quantitatively.

Spectral imaging is a new technology by which the signal light of detected sample is divided into several narrow bands, and then the images at each band are captured by the detector in sequence. Both the spatial data and the spectra data can be obtained simultaneously. Spectral imaging technology has been applied in many fields such as food safety [12], fruit quality [13, 14], and medicine testing [15, 16]. In this paper, fluorescence spectral imaging method was used to work on the formation ratio of agarwood.

## 2. Materials and Methods

### 2.1. Sample Preparation

The tested samples were cut from seven-year-old* Aquilaria sinensis* at height of 1.3 meters above the ground.* Aquilaria sinensis* grew in an artificial forest in Huazhou, Guangdong province, China, and were treated by liquid transfusion technology of agarwood formation in the whole body and then were cut two years later. The samples were dried, polished to flat surface, and then stored at 25°C ± 3 before measuring.

### 2.2. Testing System

The testing system was designed in Guangdong Key Laboratory for Innovative Development and Utilization of Forest Plant Germplasm, South Agricultural University. The main components of the system are two 365 nm UV lamps (EA-160/FA, Spectronics Corp., NY, USA), optical filters (300 nm–800 nm, Thorlabs Inc., NJ, USA), a CCD (1/2′′, 1280 × 1024) and camera lens (M0814-MP, Computer Company, Japan), and a host computer.

**Figure 1:**
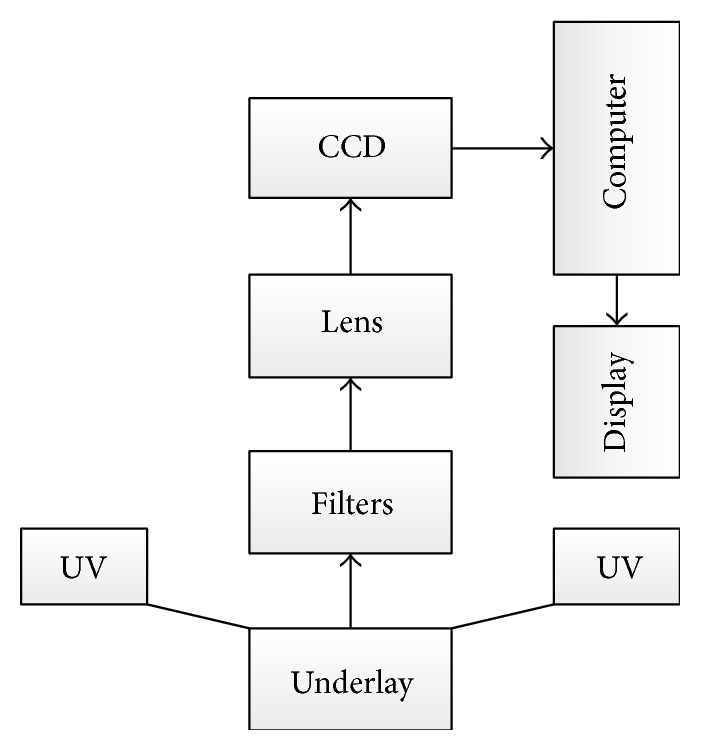
The fluorescence spectral imaging system.

The two UV lamps are used as excitation light source which could excite sample plan uniformly. The optical filters are used to select the wavelengths of emission signals of samples. The working wavelength range is from 450 nm to 800 nm with the interval 10 nm. The spatial resolution is 5 lp/mm. The ray path of the system was shown in Figure 1. The UV light coming from the light source reached the samples on the underlay, and then the samples were stimulated to emit fluorescence light. The emission light was filtered by the filters and captured by the CCD. At last, a spectral cube of samples was got and analyzed. Each sample has one spectral cube.

### 2.3. Data Analysis

**Figure 2:**
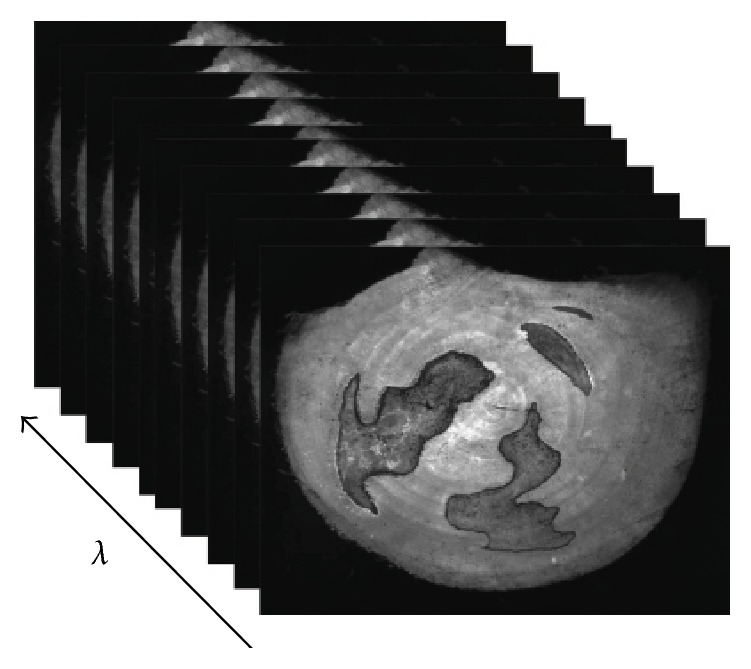
Spectral cube of a sample.

A spectral cube is shown in Figure 2, which consisted of 16 spectral images. The pictures in the same cube are spatially matched, but spectrally different.

There are four parts in each picture, nonagarwood, agarwood, rotten wood, and the background. Since the spatial distribution of nonagarwood, agarwood, and rotten wood is irregular, the spectra differences of these parts were invited to determine the spatial position pixel by pixel. Here the pictures whose wavelength was between 500 nm and 650 nm were used because the spectral differences were the biggest in this wavelength range. There are four steps to analyze data here.

#### 2.3.1. Obtaining the Spectral Curve of Each Part

For positioning the spatial distribution of nonagarwood, agarwood, and rotten wood in one sample, the spectral cube of the sample should be analyzed to get the spectral curve of each part. As an example, to get the spectral curve of agarwood, first, the mask was designed based on prior knowledge for choosing a 3 × 3 area of nonagarwood on one picture of the spectral cube; second, the average intensity of chosen area was calculated, which could represent the intensity of nonagarwood at the wavelength of this picture; third, the same areas of other pictures were analyzed like the first and second steps to get a group of intensities at different wavelength, which is the spectral curve data of the agarwood. The spectral curve of the nonagarwood and the rotten wood was also obtained by the same way.

#### 2.3.2. Obtaining the Outline of the Sample

The outline of the whole sample is needed for getting the relative area of the whole sample. To obtain the outline of the sample, a high-pass filter [17, 18] in the spatial domain had been used, which was shown in the following expresion:

(1) $$\begin{array}{c} I=\left\{\begin{array}{cc} I & \left(I\geq A\right)\\ 0 & \left(I<A\right), \end{array}\right. \end{array}$$

in which *I* is the intensity of a pixel and *A* is the cutoff intensity. The signal which is higher than or equal to *A* could pass the filter, but the signal which is lower than *A* would be attenuated.

The filter shown in expression (1) was used to scan the spectral image line by line from left side. The scanning would not stop until the first pixel which could pass the filter appears. The pixel is the left edge of the sample in corresponding line. Then the filter begins second scanning from right side to look for the right edge.

#### 2.3.3. Distinguishing Nonagarwood and Rotten Wood

Distinguishing agarwood directly from sample is difficult here, so the nonagarwood and rotten wood were distinguished, respectively, at first. Which wavelengths were selected was based on the differences of spectral curve of these three parts. Edge detection filter [19–22] was designed to detect the edge of nonagarwood. The edge detection filter worked with four steps. First, smooth the picture to reduce the noise by Gaussian filter. Second, calculate the local gradient and the edge direction of each spot to get the ridges with the following expression:

(2) $$\begin{array}{c} g\left(x,y\right)={\left[G_{x}^{2}+G_{y}^{2}\right]}^{1/2},\\ \alpha \left(x,y\right)=\arctan\left(\frac{G_{y}}{G_{x}}\right). \end{array}$$

Here, *g*(*x*, *y*) is the local gradient of each point, *G*_*x*_ is the partial derivative of pixel (*i*, *j*) in direction *x*, and *G*_*y*_ is the partial derivative of pixel (*i*, *j*) in direction *y*. *a*(*x*, *y*) is the direction of the edge. The edge point is defined as the largest gray value point of the local area on the gradient direction. All these edge points could form the ridges of the gradient magnitude. Third, track the top of all the ridges and let all the pixels that are not on the top of the ridges be zero, which was known as nonmaximum suppression processing. Fourth, define the effective range of gray value of the ridges based on the tested sample. Here, range [0.2,0.25] was used. Every 8 connection ridge pixels in the range were integrated and linked into the strong ridge pixels, which were the edge between nonagarwood and agarwood.

The band ratio algorithms were used for rotten wood distinguishing. Band ratio is created by dividing spectral values of one band by spectral values of another band from a spectral cube. It is used to enhance the spectral differences between bands and to reduce the effects of topography [23, 24]. Here, band ratio algorithms were used to enhance the differences between the rotten wood and the other part of the sample. The band ratio method is expressed in

(3) $$\begin{array}{c} BV_{i,j,r}=\frac{BV_{i,j,k}}{BV_{i,j,l}}, \end{array}$$

in which *V*_*i*,*j*,*k*_ and *V*_*i*,*j*,*l*_ are the gray value of the pixel (*i*, *j*) on *k* band and *l* band pictures and *BV*_*i*,*j*,*r*_ is the ratio value of the pixel (*i*, *j*). If the denominator of the expression is zero, *BV*_*i*,*j*,*r*_ will be assigned to zero.

To express the range of function (3) by linear fashion and use the standard 8-bit encodings (range from 0 to 255), normalized function should be used to maintain further processing, and the following are the formulas:

(4) $$\begin{array}{c} BV_{i,j,n}=\left\{\begin{array}{cc} 0 & BV_{i,j,r}=0\\ \text{Int}\left[\left(BV_{i,j,r}*127\right)+1\right] & BV_{i,j,r}\in \left[\frac{1}{255},1\right]\\ \text{Int}\left(\frac{BV_{i,j,r}}{2}+128\right) & BV_{i,j,r}\in \left(1,255\right], \end{array}\right. \end{array}$$

in which *BV*_*i*,*j*,*n*_ refers to the output gray value of the pixel (*i*, *j*) and the “Int” refers to integer conversion.

After the enhanced image by band ratio algorithms was proceeded by the edge detection filter mentioned, the edge between agarwood and rotten wood was obtained.

#### 2.3.4. Obtaining the Agarwood Formation Ratio

Since the area of no rotten wood and the area of no nonagarwood are obtained, for obtaining the agarwood formation ratio, two steps are needed, obtaining the edge of the agarwood and connecting the region. Function (5) was used to obtain the edge:

(5) $$\begin{array}{c} C=A\cap B, \end{array}$$

in which *A* and *B* refer to the two images which were got in Section 2.3.3. *C* refers to the edge of agarwood. The edge of agarwood could be got by intersecting the complementary sets of nonagarwood and rotten wood area. For getting the area of agarwood, the region surrounded by the edge should be connected. The edge just like a ring which has two sides. Track the outside of the edge and the corresponding inside of the edge, and then connect both sides to get the agarwood area.

## 3. Results

### 3.1. Spectral Curve

**Figure 3:**
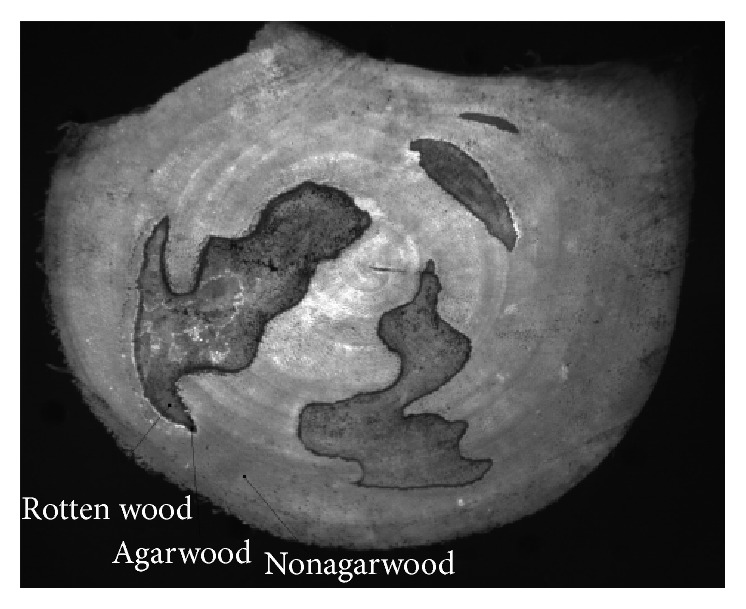
The spatial distribution of nonagarwood, agarwood, and rotten wood.

**Figure 4:**
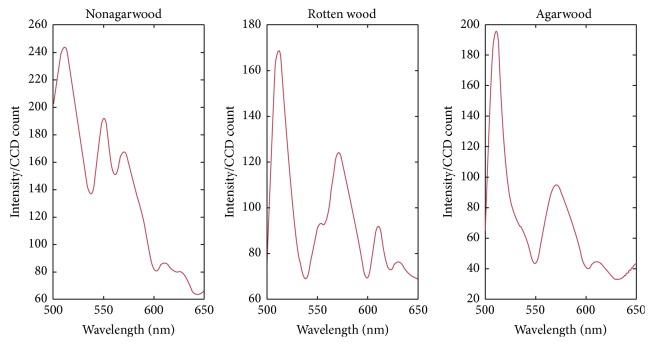
The spectral graphs of nonagarwood, rotten wood, and agarwood.

The picture at 550 nm was used to select the area of agarwood, nonagarwood, and rotten wood. The selected areas were shown in Figure 3. The three spectral curves were shown in Figure 4. The differences of three spectral curves were obvious by analyzing Figure 4. The fluorescence intensity at each wavelength of nonagarwood is much higher than that of agarwood and rotten wood. Here, the picture at 550 nm was used. Comparing the spectra of agarwood and rotten wood, both of them had peaks at 510 nm and 570 nm. The peak of agarwood at 510 nm is higher than that of rotten wood. However, the peak of agarwood at 570 nm is lower than that of rotten wood. So the band ratio of 510 nm and 570 nm was used to enhance the differences of rotten wood and agarwood.

### 3.2. The Outline of the Sample

**Figure 5:**
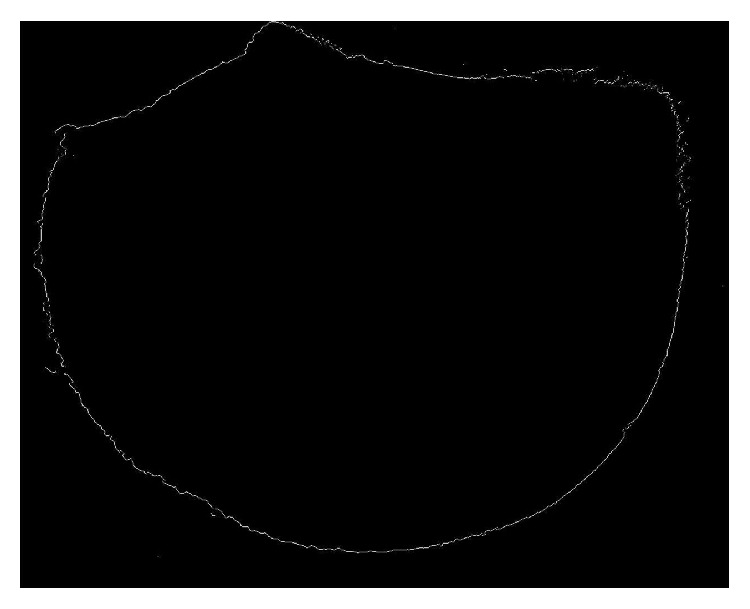
The outline of the sample.

The outline of the sample was shown in Figure 5, which was got by high-pass filter. Comparing Figure 5 with Figure 3, the outline of the sample had been got accurately.

### 3.3. The Area Ratio of Agarwood

**Figure 6:**
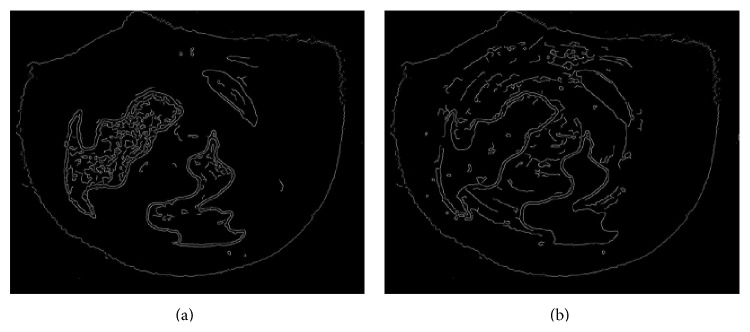
(a) Exclude the nonagarwood; (b) exclude the rotten wood.

The results of distinguished nonagarwood and rotten wood were shown in Figure 6. In Figure 6(a), the area of nonagarwood was all in the dark, but the area of the agarwood and rotten wood was filled by bright pixels. In Figure 6(b), the area of rotten wood was all in the dark, but the area of the agarwood and nonagarwood was full of bright pixels. The common area of bright pixels in two pictures is the area of agarwood.

**Figure 7:**
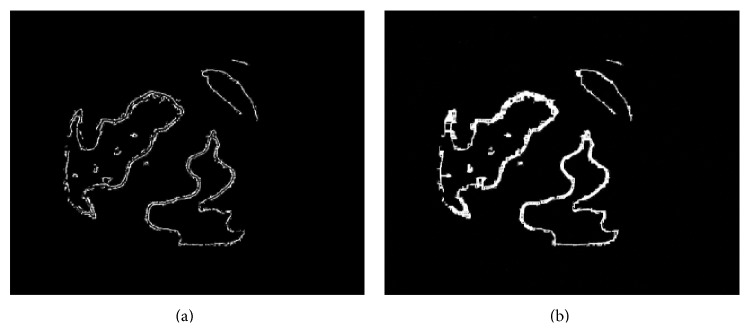
(a) The intersection of nonagarwood and rotten wood; (b) the area of the Chinese agarwood.

The result got by intersecting Figures 6(a) and 6(b) was shown in Figure 7(a). The edges of the agarwood were tracked accurately. Filling the area between the inside and outside edges, the agarwood could be got, which was shown in Figure 7(b). The results are consistent with the judgment made by the expert in traditional Chinese medicines [25, 26]. The relative area of agarwood could be got by counting the number of bright pixels in Figure 7(b).

Counting the number of pixels surrounded by the outline, the relative area of the whole sample could be got. The area percentage of agarwood is the ratio of relative area of agarwood to relative area of the whole sample. For the sample showed in the paper, the area percentage of agarwood is 2.06%.

## 4. Discussion

**Figure 8:**
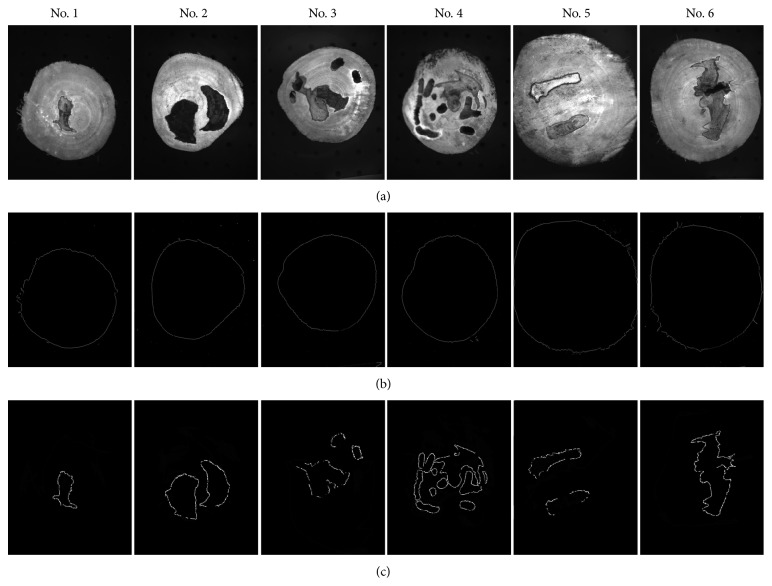
Agarwood formation ratio of six samples.

35 samples were analyzed by fluorescence spectral imaging technique to evaluate the validity of the method. Six of them were shown in Figure 8. Line (a) is spectral images at 550 nm, Line (b) is the outlines of samples, and Line (c) is the spatial distribution of agarwood in samples. The area percentage of agarwood of these six samples was shown in Table 1. The results show that the technique works well on detecting the agarwood formation ratio.

The fluorescence spectral imaging technique tested samples pixel by pixel by the spectral information, which had been applied successfully to test spatial distribution of the constituents in traditional Chinese medicines by our group [16, 27]. Spatial distribution of agarwood was detected by fluorescence spectra of agarwood in this paper. Before the fluorescence spectral imaging technique was presented, the agarwood only can be judged manually. The experts could point out the distribution of agarwood but could not provide quantitative data. The new technique can test formation ratio quantitatively. The validity of the results was proved by the expert in field of identification of Chinese medicine.

## 5. Conclusions

The agarwood which formed in* Aquilaria sinensis* was measured by fluorescence spectral imaging technique in this paper. The agarwood formation ratio is an important factor to indicate the better trees, better liquid transfusion, and better planting technique for agarwood formation. To our knowledge, this is the first time that the agarwood formation ratio was measured quantitatively. Comparing to qualitative estimation by manual watching, the technique is much more accurate. It is concluded that fluorescence spectral imaging is a precise, noninvasive, and fast technique for measuring agarwood formation ratio and quality control of agarwood cultivation.

## Figures and Tables

**Table 1:** Agarwood formation ratio of six samples.

No. 1	No. 2	No. 3	No. 4	No. 5	No. 6
0.48%	1.24%	0.68%	2.45%	0.44%	0.90%
